# The Insular Cortex Dynamically Maps Changes in Cardiorespiratory Interoception

**DOI:** 10.1038/npp.2017.154

**Published:** 2017-08-09

**Authors:** Mahlega S Hassanpour, W Kyle Simmons, Justin S Feinstein, Qingfei Luo, Rachel C Lapidus, Jerzy Bodurka, Martin P Paulus, Sahib S Khalsa

**Affiliations:** 1 Laureate Institute for Brain Research, Tulsa, OK, USA; 2 Oxley College of Health Sciences, University of Tulsa, Tulsa, OK, USA; 3 Department of Psychology, University of Tulsa, Tulsa, OK, USA; 4 Stephenson School of Biomedical Engineering, University of Oklahoma, Norman, OK, USA

## Abstract

Palpitations and dyspnea are fundamental to the human experience of panic anxiety, but it remains unclear how the brain dynamically represents changes in these interoceptive sensations. We used isoproterenol, a rapidly acting peripheral beta-adrenergic agonist similar to adrenaline, to induce sensations of palpitation and dyspnea in healthy individuals (*n*=23) during arterial spin labeling functional magnetic resonance imaging (fMRI). We hypothesized that the right mid-insular cortex, a central recipient of viscerosensory input, would preferentially respond during the peak period of cardiorespiratory stimulation. Bolus infusions of saline and isoproterenol (1 or 2 μg) were administered in a blinded manner while participants continuously rated the intensity of their cardiorespiratory sensation using a dial. Isoproterenol elicited dose-dependent increases in cardiorespiratory sensation, with all participants reporting palpitations and dyspnea at the 2 μg dose. Consistent with our hypothesis, the right mid-insula was maximally responsive during the peak period of sympathetic arousal, heart rate increase, and cardiorespiratory sensation. Furthermore, a shift in insula activity occurred during the recovery period, after the heart rate had largely returned to baseline levels, with an expansion of activation into anterior and posterior sectors of the right insula, as well as bilateral regions of the mid-insula. These results confirm the right mid-insula is a key node in the interoceptive network, and inform computational models proposing specific processing roles for insula subregions during homeostatic inference. The combination of isoproterenol and fMRI offers a powerful approach for evaluating insula function, and could be a useful probe for examining interoceptive dysfunction in psychiatric disorders.

## Introduction

Palpitations and dyspnea are fundamental symptoms of panic anxiety (Craske *et al*, 2010). It has been suggested that altered processing of these interoceptive sensations contributes to the development and/or maintenance of panic and other anxiety disorders (Bouton *et al*, 2001; Domschke *et al*, 2010; Paulus and Stein, 2010). While it is clinically well documented that individuals with panic disorder report prominent sensitivity toward acutely elevated interoceptive sensations (Boettcher *et al*, 2015), and that they disproportionately panic during their pharmacological induction (Balon *et al*, 1990; Pohl *et al*, 1988), a limited knowledge of the neuroanatomical structures recruited during cardiorespiratory interoceptive processing hinders progress in understanding the underlying neurobiological mechanisms.

Animal and human studies have suggested that the insular cortex has a central role in the integration and representation of cardiorespiratory and other interoceptive signals (Cameron, 2009; Craig, 2002; Oppenheimer and Cechetto, 2016; Saper, 2002). Functional neuroimaging studies have consistently revealed the insula to be a key viscerosensory region, and it is commonly considered to be the principal cortical target receiving information about interoceptive body states (Craig, 2009; Critchley and Harrison, 2013). However, other sensory regions, including the somatosensory cortices, have also been implicated in cardiorespiratory interoception (Cameron and Minoshima, 2002; Kern *et al*, 2013; Khalsa *et al*, 2016; Khalsa *et al*, 2009a), raising questions about the extent to which the insula has a singular role. Importantly, the insula exhibits unique cytoarchitectural diversity, with a six-layered granular/hypergranular isocortex in the dorsal posterior region, a four-layered agranular ventral anterior region, and an intermediary dysgranular middle region. These subregions are known to have differing connections to a variety of sensory regions across the brain (Chikama *et al*, 1997; Mesulam and Mufson, 1982), and differential resting-state functional connectivity (Kurth *et al*, 2010; Sporns, 2014; Uddin, 2015) suggesting functional specificity within the insula.

To examine the cortical mapping of acute elevations in cardiorespiratory sensation we have used bolus infusions of isoproterenol, a rapid beta-adrenergic agonist similar to adrenaline that targets peripheral beta-adrenergic receptors in heart and lungs (Khalsa *et al*, 2016). During our initial functional magnetic resonance imaging (fMRI) study in healthy individuals, we concurrently measured arterial and venous activity using arterial spin labeling (ASL) and blood-oxygenation-level-dependent (BOLD) signals. We observed dose-related increases in BOLD activation of the right mid-insula and posterior insula during the peak heart rate response, consistent with a sensory afferent processing role (Hassanpour *et al*, 2016), and activation in more anterior portions of the insula during the anticipatory period, before the onset of isoproterenol action, consistent with a predictive processing role (Barrett and Simmons, 2015; Seth and Friston, 2016; Stephan *et al*, 2016). We also observed ASL activations in the same region of the mid-insula, albeit to a lesser extent, as the cluster did not survive cluster size correction for multiple comparisons. We speculate that this absence of significant hemodynamic activity on the arterial side of the vascular bed was related to the intrinsically low signal-to-noise ratio of ASL compared to BOLD, as well as to the low experimental statistical power associated with using only one infusion trial per dose. Another limitation of the previous study was the lack of real-time sensation measurements. That is, sensation reports were recorded retrospectively after participants exited the scanner, limiting the ability to characterize different stages of cardiorespiratory sensory experience.

The current experiment was designed to address these limitations of the previous study while expanding the scope of analysis. For this experiment, we focused our recording on using only ASL during isoproterenol administration, to verify whether we could replicate the mid-insula signal using an improved experimental design with increased statistical power. ASL is preferred in pharmacological studies because it provides a quantitative measure of cortical physiology (Wang *et al*, 2011) and additionally it is more localized to the parenchyma than BOLD (Kim and Ogawa, 2012). To improve our assessment of subjective interoceptive experience we added continuously recorded ratings of cardiorespiratory sensation during each infusion using a MRI-compatible dial. To judge the individual impact on sensory and affective processes we acquired retrospective ratings of cardiac, respiratory, negative, and positive valence experiences in the scanner immediately following the end of each infusion. To identify neural responses during different stages of cardiorespiratory processing, we examined activation across the brain during the peak heart rate response period and also during the subsequent period of recovery once heart rate changes had largely resolved. We hypothesized that (1) the right mid-insula would respond preferentially during the peak period of stimulation and (2) activation patterns would propagate into adjacent territories of the insula during the recovery period.

## Materials and methods

### Participants

A total of 23 healthy unmedicated individuals (22 right-handed, 11 female; mean age: 26±6 years; body mass index: 25.9±4.3) participated in this study. The study was approved by the Western Institutional Review Board and conducted at the Laureate Institute for Brain Research. All participants provided written informed consent and received compensation for their participation (see Supplementary Methods for complete details).

### Experimental Protocol

To parametrically modulate cardiorespiratory sensations and quantitatively measure the brain’s hemodynamic response to these changes, participants received intravenous bolus infusions of isoproterenol hydrochloride (1 or 2 μg per dose; Valeant Pharmaceuticals, Laval, QC, Canada) during an ASL imaging session. Bolus infusions of normal saline were used as a control condition. Each dose (1 μg, 2 μg, and saline) was repeated twice, resulting in a total of six infusion scans. Infusions were administered in a double-blinded manner at 60 s into each scan, and infusion order was randomized across subjects. Throughout each infusion scan (240 s/scan), participants continuously rated their experience of cardiorespiratory sensation intensity by rotating a MRI-compatible dial (Current Designs, Philadelphia, PA, USA) clockwise or counterclockwise, with their dominant hand, when the sensations increased or decreased. They were instructed to keep their eyes open during the scans, and their dial ratings were displayed in real time on a computer screen in front of them (Supplementary Figure S1). Concurrent with ASL recording, cardiac and respiratory waveforms were acquired at 40 Hz using a MRI-scanner-equipped pulse oximeter and a respiratory transducer belt, and two MRI-compatible ECG leads (lead I and II configuration) were attached for continuous cardiac rhythm safety monitoring (GE Healthcare, Waukesha, WI, USA). Infusions were delivered by a nurse seated inside of the scanner room, with continuous visual access to all monitored vital signs. Immediately after each infusion scan, subjects were asked to verbally rate the intensity of experienced heartbeat and breathing sensations (0=‘none or normal’ and 10=‘most ever’). To index affective state participants also reported the intensity of experienced positive emotion (happy, excited, or euphoric) and negative emotion (anxious, tense, or nervous) (see Supplementary Methods for detailed instructions).

### MRI Data Acquisition

Experiments were performed on a 3 T General Electric (GE) MR750 MRI scanner (GE Healthcare) with an 8-channel receive-only head coil. Head movement was minimized by securing the head with soft foam padding, and by lightly affixing a piece of soft tape to the participant’s forehead. Functional images of 24 axial interleaved slices were acquired using a pseudo-continuous ASL (pCASL) sequence (see Supplementary Methods for complete details).

### Data Analysis

#### Pre-processing

For each run, all functional volumes were co-registered to the first control volume and then spatially smoothed using a 3D Gaussian kernel with full width half maximum of 6 mm in AFNI (Cox, 1996). The first four volumes were discarded. Time-locked fluctuations in the respiratory and cardiac frequencies and their first harmonics were removed from the ASL signal using the RETROICOR procedure (Restom *et al*, 2006) applied separately on tag and control images using a custom code in MATLAB (MathWorks, Natick, MA, USA). Perfusion images were calculated using pair-wise subtraction, and CBF values were calibrated. ASL calibration images were also corrected for sensitivity and then normalized to the standard Talairach atlas (TT_Daemon) to calculate the mean CSF signal within the ventricles to be used as a measure of the equilibrium magnetization of arterial blood. CBF in each voxel was then calculated using a general kinetic model for pCASL (Alsop *et al*, 2015). CBF images were then spatially transformed to the Montreal Neurological Institute 152 atlas space using an affine transformation in FSL (Jenkinson *et al*, 2002) (see Supplementary Methods for complete details).

#### Statistical analysis

Subject-level maps of the brain response to different stages of cardiorespiratory stimulation were generated separately for each infusion using a block averaging method. On the basis of the experimental design, and our previous observations of different stages of group-averaged heart rate changes and subjective dial ratings during isoproterenol infusions (Hassanpour *et al*, 2016; Khalsa *et al*, 2015; Khalsa *et al*, 2016; Khalsa *et al*, 2009b), we defined three time-course blocks/periods: baseline (0−60 s); peak (80−140 s); and recovery (160−240 s). Contrast maps for the peak (and separately, recovery) *vs* baseline periods were generated by subtracting the average of all the volumes within baseline blocks from those within peak (and separately, recovery) blocks. Next, group-level statistical maps of brain activation were generated by averaging the contrast maps across subjects and estimating the variance using a random effects analysis. Statistical maps were thresholded at *p*<0.005 (uncorrected). A cluster size analysis based on random field theory was performed to determine the statistical significance of above threshold cluster level family-wise error at *p*<0.05 (corrected).

## Results

**Figure 1:**
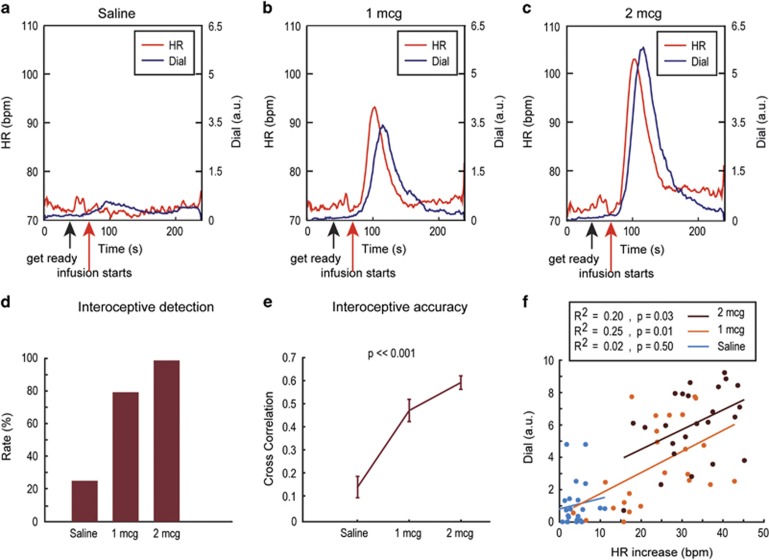
(a–c) Group averages of continuous heart rate (red curve) and cardiorespiratory intensity ratings (blue curve) during saline, 1 μg, and 2 μg isoproterenol infusions. (d) Cardiorespiratory detection rates at different doses. (e) Zero-order cross correlation between interoceptive dial rating and heart rate response. Error bars indicate s.e.’s. (f) Maximum cardiorespiratory intensity dial ratings *vs* heart rate increases during peak period for saline (blue), 1 μg (orange), and 2 μg (dark brown), along with the corresponding *R*-squares and associated *p*-values for linear regression. b.p.m., beats per minute; HR, heart rate.

Real-time ratings of cardiorespiratory sensations at each dose tended to follow the observed changes in heart rate (Figure 1a–c; Supplementary Figure S2), revealing that cardiorespiratory sensations were detected in a dose-dependent manner (Figure 1d). While a minority of participants reported increased sensations during saline infusions (Figure 1a and d), the majority of participants reported increased sensations during the 1 μg dose (Figure 1b and d), and all participants reported increased sensations during the 2 μg dose (Figure 1c and d). Interoceptive accuracy, measured by calculating the zero-order cross correlation (with no temporal shift) between interoceptive dial ratings and heart rate responses, significantly increased across isoproterenol dose (Figure 1e). A significant positive correlation between the heart rate increase and maximum dial rating during the peak period was observed at 1 μg (*p*=0.01) and 2 μg (*p*=0.03) isoproterenol but not saline (*p*=0.50) infusions (Figure 1f), indicating good correspondence between changes in cardiac signal and subjective experience. We did not find significant correlations between respiratory changes and maximum dial ratings during isoproterenol infusions (Supplementary Figure S2).

**Figure 2:**
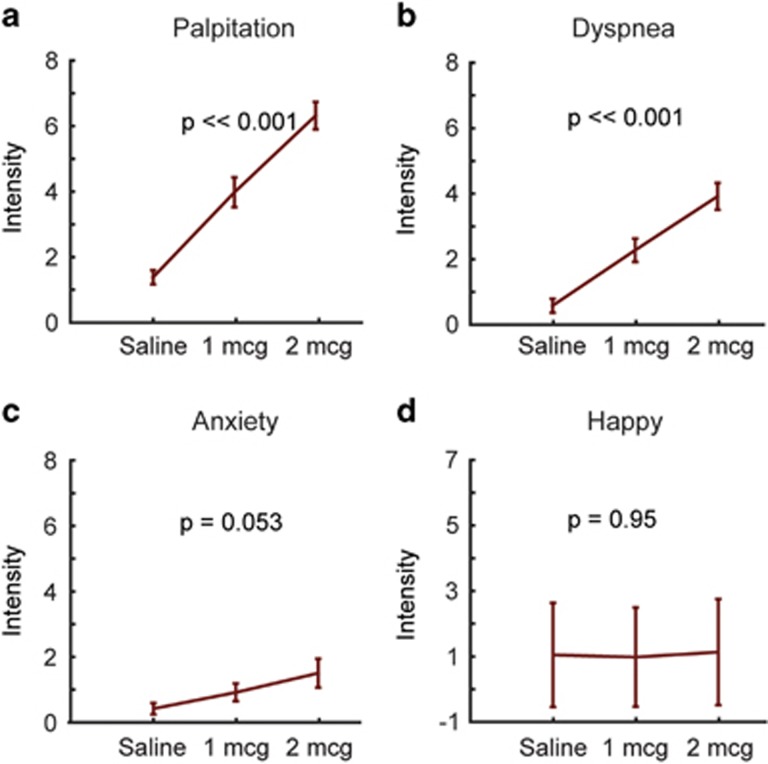
Retrospective ratings of (a) palpitations, (b) dyspnea, (c) negative emotion, and (d) positive emotion experienced at different doses. Error bars show s.e.’s. *p*-values for one-way ANOVA analysis of dose effect are reported.

Participants reported significant dose-related increases in retrospective reports of palpitation (F=45.4, df=66, *p*<<0.001) and dyspnea (F=26.1, df=66, *p*<<0.001; Figure 2a and b, respectively). We observed a trend toward a dose-related increase in negative emotion ratings (F=3.7, df=66, *p*=0.053) but no effect on positive emotion ratings (F=0.05, df=63, p=0.95; Figure 2c and d, respectively).

**Figure 3:**
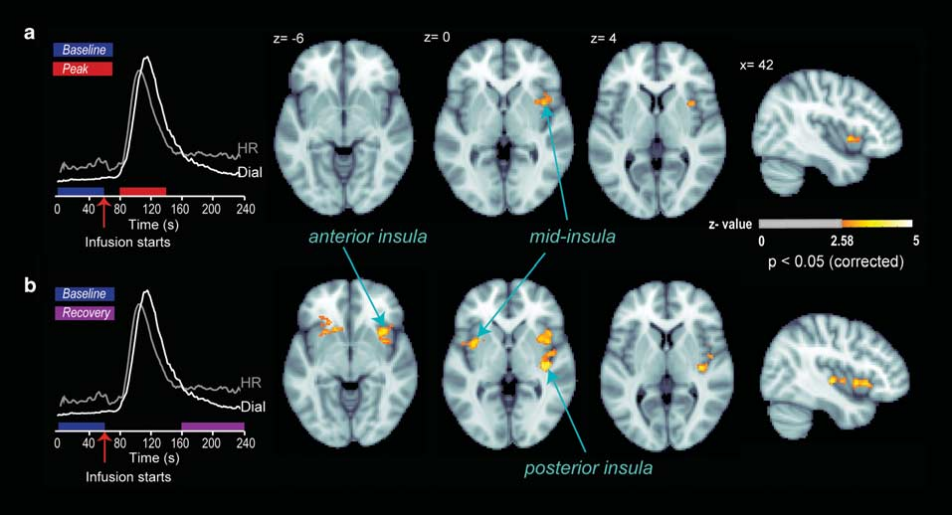
Cortical activity during different stages of cardiorespiratory interoceptive processing following 2 μg isoproterenol infusion. (a) A voxelwise whole-brain analysis showed an increase in right mid-insula activity during the peak period relative to the baseline period (shown with the red and blue bars on the left side of the plot). (b) A voxelwise whole-brain analysis showed bilateral increases in insula activity during the recovery period (shown with the purple bar), at multiple subregions, including the right and left mid-insula, the right anterior insula, and the right posterior insula.

**Figure 4:**
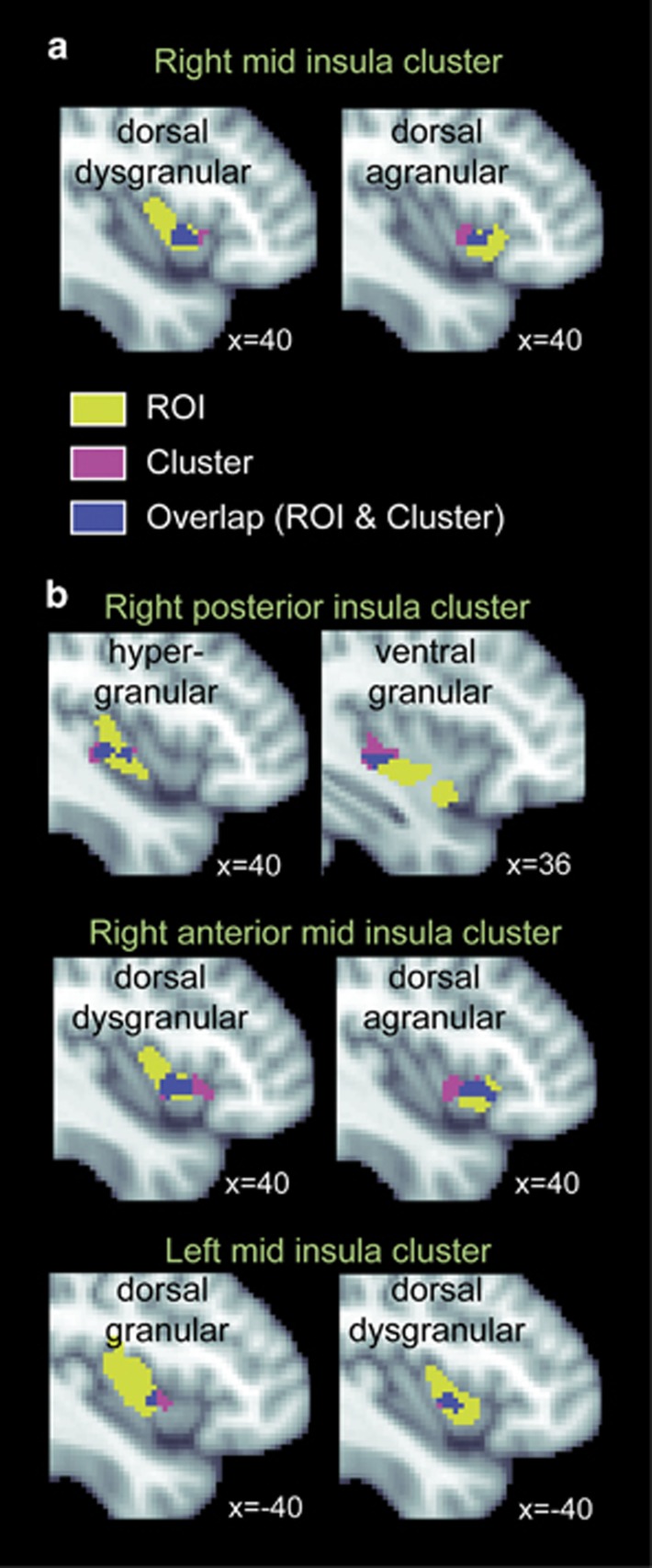
Insula activations for the peak and recovery periods displayed against the backdrop of all relevant probabilistic cytoarchitectonic insula subregions (thresholded at 25% probability, as per Fan *et al* (2016)). (a) During the peak period, the cluster in the right mid-insula spans mostly the dorsal dysgranular insula. (b) During the recovery period, the cluster in the right posterior insula spans mostly the hypergranular insula, the cluster in the right anterior mid-insula spans both the dorsal dysgranular and agranular insula, and the cluster in the left mid-insula spans mostly the dorsal dysgranular insula (see Supplementary Table 2 for a breakdown of the percentage overlap for each cluster with each subregion).

We focused our analysis of cerebral perfusion data on the 2 μg dose, as this was the only dose for which all participants detected changes in cardiorespiratory sensations (similar to Hassanpour *et al* (2016)). Consistent with our first hypothesis, a voxelwise analysis of the CBF data across the entire brain revealed a significant increase in activity in a cluster of voxels located in the right mid-insula during the peak period (voxelwise threshold at *p*<0.005 and cluster size threshold at *p*<0.05 were applied; Figure 3a and 4a). We also observed a significant cluster of activation in the left medial frontal gyrus consistent with premotor cortex (Supplementary Table S1). Inspection of the dial recordings during the recovery period showed that subjects continued to report experiencing changes in cardiorespiratory sensations, even after the heart rate had reached a steady state (ie, note that in right third of Figure 2c the blue ‘dial’ line continues to change substantially even after the red ‘heart rate’ line reaches a steady state at ~75 b.p.m.). During this recovery period, we observed significant activations in the anterior and posterior parts of the right insula, as well as the homologous region of the left mid-insula (Figure 3b and 4b; Supplementary Tables 1 and 2). Applying the same voxelwise whole brain analysis to the saline and 1 μg infusions did not reveal any significant clusters of activation in the insula or other somatosensory brain regions.

We also computed exploratory correlational analyses between brain activations at the 2 μg dose and the associated state interoceptive intensity ratings, state anxiety ratings, a trait interoceptive self-report measure (Mehling *et al*, 2012), and trait anxiety measures (Spielberger *et al*, 1983). ASL activity in the insula did not correlate significantly with any of these measures.

Finally, we evaluated whether implementing additional non-synchronized physiological noise reduction methods would (a) enhance or (b) eliminate CBF maps of the brain’s response to cardiorespiratory stimulation (Hassanpour *et al*, 2017). Our analysis revealed a substantial increase in the volume of activated insula subregions during the peak period and to a lesser extent during the recovery period, strongly supporting the former possibility (Supplementary Figure 3; Supplementary Tables 3 and 4; see Supplementary Results for complete details).

## Discussion

The present study demonstrates that the cerebral blood flow response across human insula subregions varies dynamically as a function of the actual and perceived cardiorespiratory responses to sympathetically induced homeostatic perturbation. First, we replicated with ASL fMRI our previous BOLD fMRI finding of increased cortical activity in the right mid-insula during peripheral adrenergic stimulation with isoproterenol (Hassanpour *et al*, 2016), confirming that this region dynamically tracks sympathetic cardiorespiratory interoception during homeostatic deviations of body state. More importantly, however, we demonstrated for the first time that neural activity shifts to other insula subregions, including the right anterior, right posterior, and left mid-insula during the subsequent recovery stage, when cardiorespiratory signals have reached a steady state.

The novel evidence presented here that the locus of insula activity shifts dynamically with peripheral stimulation and awareness suggests important processing roles for insula subregions during recovery from homeostatic perturbation. These roles are directly relevant to prominent theories of insula function that have emphasized different roles for subregions of the insular cortex in the central representation of interoceptive sensations. For example, the agranular anterior insula has been typically postulated as a region that ‘instantiates all subjective feelings from the body and feelings of emotion in the immediate present’ (Craig, 2009). Contrary to this view the findings from the present study, and the one preceding it (Hassanpour *et al*, 2016), directly suggest that the conscious experience of ongoing cardiorespiratory changes is tightly linked to activity of the dysgranular mid-insula. Furthermore, our results confirm that the agranular anterior and granular/hypergranular posterior insula also dynamically contribute to conscious interoceptive experience, but they do so during different temporal windows indicating a different role in the processing of interoceptive signals.

Recent views of the agranular anterior insula, which consider it to be at the top of a neural hierarchy, ascribe an active visceromotor role to this region such that interoceptive predictions are issued from it to establish homeostatic setpoints for behavioral and physiological responses (Seth and Friston, 2016; Smith *et al*, 2017; Stephan *et al*, 2016). A different model postulates that the comparison between actual and expected changes also occurs within the insular cortex but along a posterior-to-anterior gradient, and across different cell layers, with predictions issued by the agranular anterior insula, and reciprocating prediction errors (PEs) issued by the dysgranular mid and granular/hypergranular posterior insula (Barrett and Simmons, 2015). Each of these models are considered active inference models based on the assumption that bodily perception and action do not operate independently, and that ongoing sensory inputs (and predictions about them) provide a form of feedback that can modify behaviors, with advantageous or deleterious consequences. Such models have been rapidly integrated into the framework of computational psychiatry, and investigated using Bayesian statistical approaches (Paulus *et al*, 2016; Stephan *et al*, 2015).

Our observation that different insula subregions were recruited during different stages of cardiorespiratory processing can be viewed within an active inference framework of interoceptive processing. In such a framework, at the peak period of stimulation, inferences (ie, the relative weight of prior expectations and predictions) would be minimized in the face of intense afferent cardiorespiratory input, resulting in minimal PE. In this dynamic setting there are multiple change points ensuing after (1) onset of isoproterenol-induced effects, (2) achievement of a temporary maximal steady state (peak change), (3) a decline of isoproterenol-induced effects, and then (4) a stable steady-state period where no bodily change occurs. While we could imagine different instances of increased or decreased PEs occurring at each change point (especially at 1), we have overly simplified the picture by examining brain activity during a broadly defined ‘peak period’ (ie, all processes in 1, 2, and 3), and ‘recovery period’ (ie, all processes in 4). We argue that PEs seem to be minimized during the peak period (especially at 2) due to the availability of ongoing bodily changes that correlate closely with reported sensation at the highest dose (eg, Figure 1e and f). Importantly, we would like to note that the current experiment cannot differentiate between the relative values of PEs potentially occurring during different phases of the experiment. (For a theoretical example in which physiological responses to an environmental perturbation are modeled at different temporal change points, see Figure 6 in Stephan *et al* (2016)). However, during the recovery period, prior information would be informed by predictions about the recent body experience (ie, peak period). Therefore, during the recovery period, which is a time when sensory input alterations have resolved (ie, heart rate is at steady state), it seems possible that current experience might be reflected by the observed dysgranular mid-insula activity, predictions might be generated by the anterior agranular insula activity, and PEs (mismatch between current experience and prediction) might be reflected by the posterior granular/hypergranular insula activity.

An alternative predictive coding account may be suggested by the EPIC model (Barrett and Simmons, 2015), which proposes that PE computations happen in cortical regions where the laminar architecture supports comparisons between bottom-up afferent interoceptive signals, and top-down interoceptive predictions. The findings of the present paper are also consistent with this account because during the recovery period, when PE signals are postulated to be elevated, we observed extensive activity in the dysgranular mid and granular/hypergranular posterior insula, regions that both have the requisite cytoarchitectonic structure to compare afferent interoceptive signals arriving via the thalamus with interoceptive prediction signals arriving via the agranular anterior insula.

A somewhat different interpretation is that the insula regions observed during the recovery period are involved in a sort of ‘visceral memory,’ ie, holding the visceral perturbation online until regulatory forces have fully brought the heart rate back down to baseline (of note, even though the heart rate had reached a steady state, it still was not yet fully down to baseline). It is also possible that the activity in the agranular anterior insula during the recovery period reflects anticipation of the next infusion and its possible consequences.

However, it is important to note that without a model of PEs that can be fitted to the induced physiological changes (eg, Seth and Friston (2016); Smith *et al* (2017); Stephan *et al* (2016)), it is difficult to decide between these different possible interpretations. The current data are not amenable to a Bayesian statistical analysis as the number of infusion trials in the current study is low by Bayesian standards, and precludes ideal model selection testing. Furthermore, the ASL signal is somewhat noisy and slow with respect to the timescales of neuronal processing, and does not allow for delineation of laminar specific activity at the current 3 T field strength. We believe that these limitations can be addressed, for instance, by using a neuroimaging approach with a higher signal-to-noise ratio (eg, BOLD), or by integrating this with multimodal methods possessing greater temporal precision (eg, electroencephalography), and/or including more trial repetitions.

**Figure 5:**
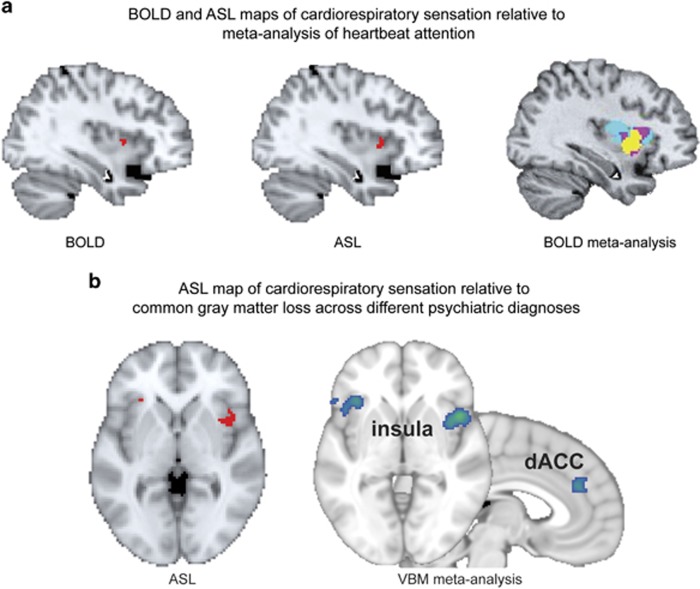
(a) Insula response to peak cardiorespiratory interoceptive stimulation from our prior BOLD fMRI study (top left), the current ASL fMRI study (top middle), and a recent meta-analysis of BOLD fMRI studies assessing attention to the heartbeat under physiological resting conditions (maximum overlap in yellow). In each case, the right mid-insula showed the most response. Reproduced with permission as follows: BOLD fMRI study (Hassanpour *et al*, 2016); and BOLD fMRI meta-analysis (Schulz, 2016). (b) Comparison between the right mid-insula response to cardiorespiratory interoceptive stimulation in the current ASL fMRI study (bottom left) and a large meta-analysis (*n*=7381 patients and 8511 matched healthy comparisons) identifying shared patterns of decreased gray matter across different psychiatric diagnoses in the same region, as well as the dorsal anterior cingulate cortex (bottom right), reproduced with permission from Goodkind *et al* (2015).

The mid-insula findings in the present study are especially noteworthy because it has been identified as the most commonly activated insula subregion across all prior studies of cardiac interoception involving directed attention to heartbeat sensations under resting physiological conditions (Schulz, 2016) (Figure 5a). Furthermore, our replication of asymmetric right insula activation during sympathetic stimulation, and hemispheric switching to include left insula activation during the recovery period, corroborates prior animal findings and a theoretical perspective positing a critical role of the right and left insula for mapping sympathetic and parasympathetic arousal, respectively (Oppenheimer and Cechetto, 2016). Taken together, these findings represent compelling evidence that the right mid-insula is a key node in the interoceptive attentional network, one that is essential for both stimulus-driven (bottom-up) and goal-directed (top-down) sympathetic viscerosensation.

Although the current study was conducted in a healthy sample, these findings have clear relevance to the search for biological underpinnings of psychiatric disorders. For instance, interoceptive abnormalities in the mid-insula have been observed in major depressive disorder (Avery *et al*, 2014; Simmons *et al*, 2016) and anorexia nervosa (Kerr *et al*, 2016). Furthermore, the current paradigm appears to precisely target the same subregion of the insula that was recently identified in a large-scale meta-analysis as showing decreased gray matter across numerous psychiatric diagnoses (Goodkind *et al*, 2015) (Figure 5b). While we cannot conclude that the observed gray matter reductions reflect an interoceptive deficit common to all psychiatric illnesses, or alternatively, that these similarities are simply coincidental, we believe that the present findings warrant further studies assessing the role of interoceptive dysfunction in psychiatric disorders (see Khalsa and Lapidus (2016) for a detailed argument).

### Limitations

There are several limitations of this study to consider. (1) The lack of mid-insula activation during the 1 μg infusion could reflect a threshold effect whereby this dose failed to adequately increase interoceptive sensations (Khalsa *et al*, 2016). Alternatively, it could reflect the low signal-to-noise ratio of ASL (Hassanpour *et al*, 2016). (2) We did not observe correlations between insula activity during exploratory analyses of the peak response period and several clinically relevant measures of anxiety and interoception. These null results do not necessarily indicate the lack of an underlying relationship and could be related to a restricted expression of psychopathology inherent to a healthy sample, the sample size not being large enough for stable correlational estimation of the population effect (Schonbrodt and Perugini, 2013), or individual difference estimates that were masked by the use of an exogenous pharmacological probe. (3) Concerns about elevated false positive rates in fMRI studies (Eklund *et al*, 2016) might potentially impact the current study given the use of a voxelwise threshold of *p*<0.005 and cluster size correction based on random field theory. Recent evidence, however, suggests these concerns may be greatly overstated (Cox *et al*, 2017). Further, we believe this is unlikely given (a) less stringent criteria could be justified due to the massive amount of *a priori* evidence that the insula is critical for interoception, and is ubiquitously activated in prior interoception neuroimaging studies, (b) that the ASL insula peak period activation replicates our previous finding with BOLD in an independent sample (Hassanpour *et al*, 2016), and (c) we performed an ROI analysis of the insular cortex, in which all insula clusters (after non-synchronized physiological noise correction) passed the cluster height threshold. We chose, however, not to focus on this ROI approach as we remain interested in exploring interoceptive relationships across the entire brain. (4) Finally, we cannot entirely rule out potential influences on the observed brain activations, such as pharmacological effects on the vasculature affecting the signal, or signal changes due to potential co-occurring changes in cerebral blood flow during respiratory stimulation. We believe these possibilities are very unlikely on the basis of our prior observations of right insula activation during cardiorespiratory interoception (Hassanpour *et al*, 2016), and based on our continued observations of insula activation after applying noise correction algorithms for synchronized (Restom *et al*, 2006) and non-synchronized physiological artifacts (Hassanpour *et al*, 2017) to the current data set (Supplementary Figure S3).

## Conclusion

The current study confirms that the right mid-insular cortex responds dynamically to peripherally mediated sympathetic elevations in cardiorespiratory interoceptive arousal, and that other insula subregions are engaged during homeostatic recovery. Collectively, this suggests that insula subregions have different processing roles in response to homeostatic deviations of body state, and that the current protocol may provide a useful probe for evaluating the role of interoceptive dysfunction in psychiatric disorders.

## Funding and disclosure

Research reported in this publication was supported by The William K. Warren Foundation, a NARSAD Young Investigator Award (SSK), and the National Institute of Mental Health Award Number K23MH112949 (SSK). The content is solely the responsibility of the authors and does not necessarily represent the official views of the National Institutes of Health. The authors declare no conflict of interest.

## Supplementary Material

Supplementary Methods

Supplementary Results

Supplementary Tables

Supplementary Figure Captions
