# Supplementary figures and images for: The Insular Cortex Dynamically Maps Changes in Cardiorespiratory Interoception
Source: Neuropsychopharmacology. 2017 Aug 9;43(2):426–34. doi: 10.1038/npp.2017.154 (PMC5729563; doi:10.1038/npp.2017.154)

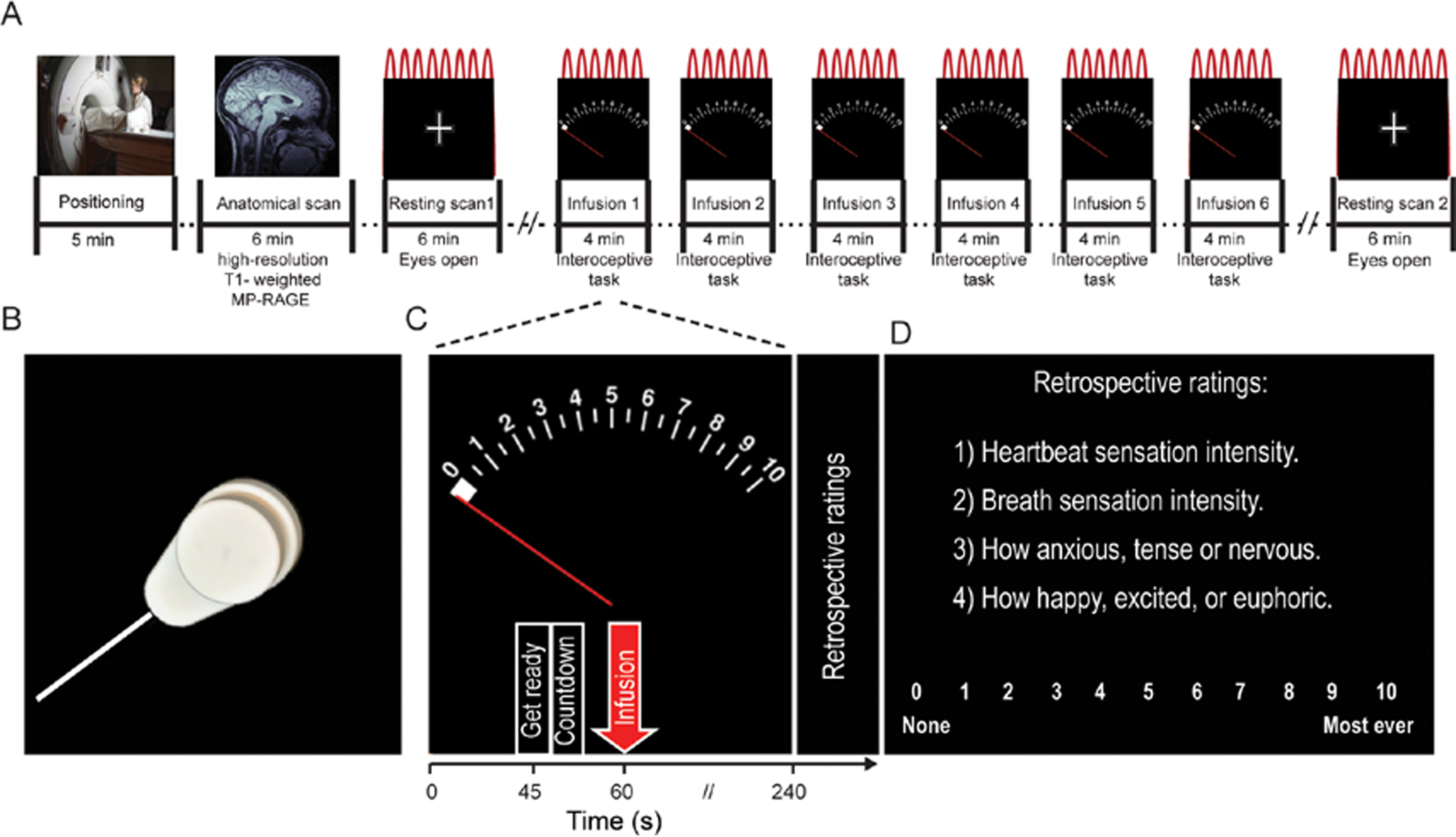

Supplement: Supplementary Figure 1 [file npp2017154x1.tif]

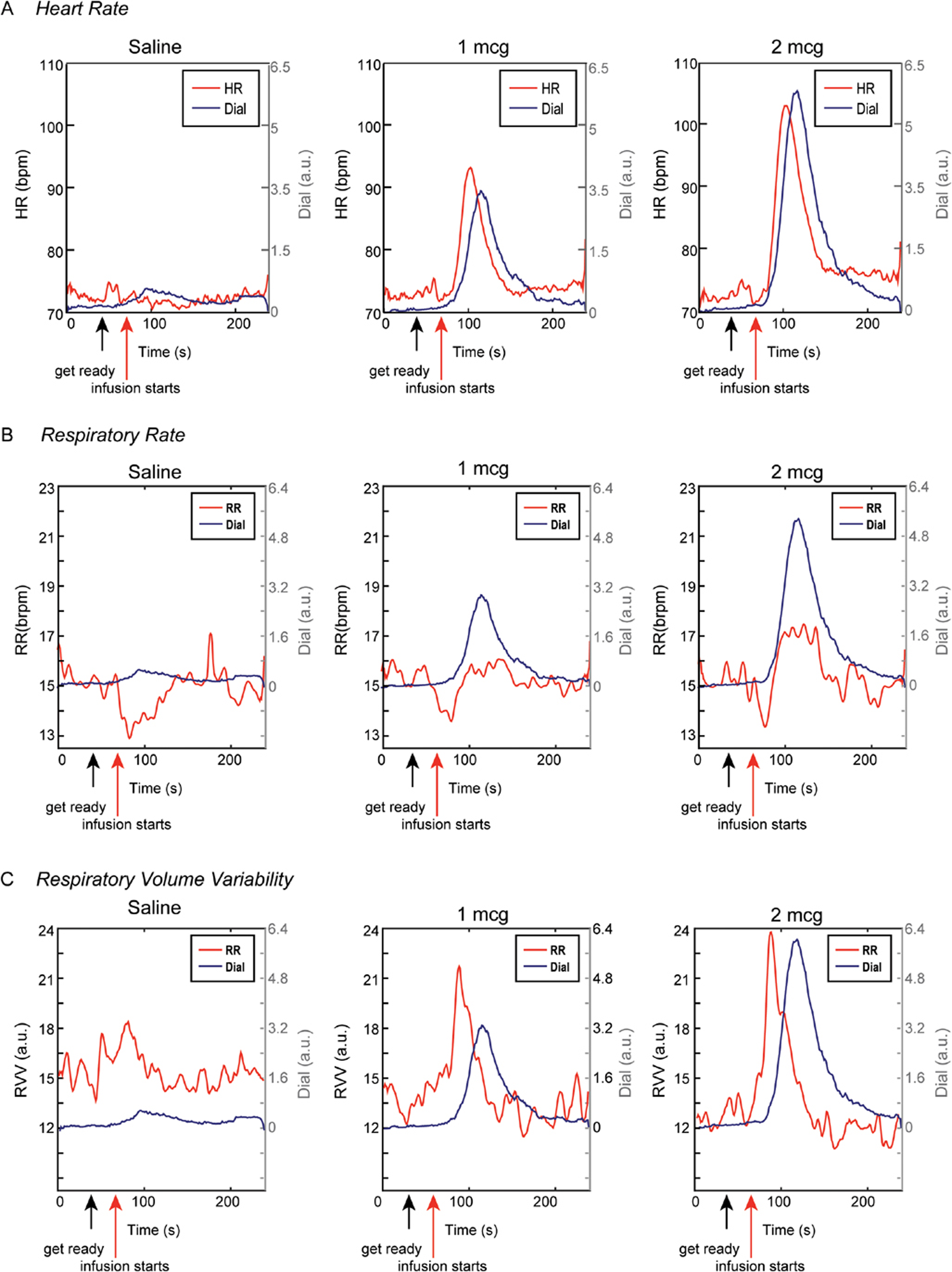

Supplement: Supplementary Figure 2 [file npp2017154x2.tif]

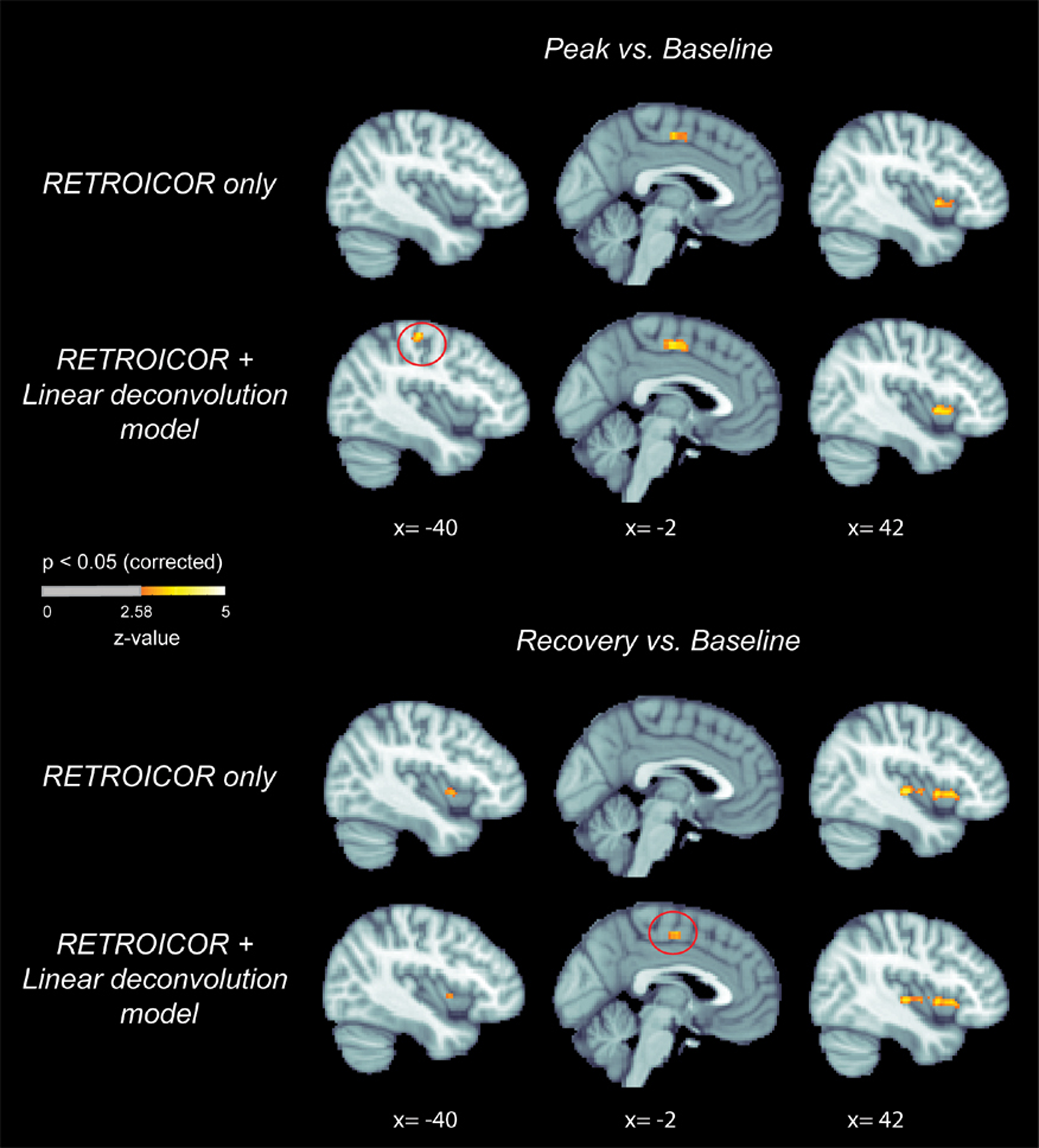

Supplement: Supplementary Figure 3 [file npp2017154x3.tif]
