# Supplementary material for: The Insular Cortex Dynamically Maps Changes in Cardiorespiratory Interoception
Source: Neuropsychopharmacology. 2017 Aug 9;43(2):426–34. doi: 10.1038/npp.2017.154 (PMC5729563; doi:10.1038/npp.2017.154)
Supplement: Supplementary Methods [file npp2017154x4.docx]

# **Full length Supplementary Methods**

# The Insular Cortex Dynamically Maps Changes in Cardiorespiratory Interoception

Mahlega S Hassanpour, PhD ^1^, W Kyle Simmons, PhD ^1,2^, Justin S Feinstein, PhD ^1,2^, Qingfei Luo, PhD ^1^, Rachel Lapidus, BA ^3^, Jerzy Bodurka, PhD ^1,4^, Martin P Paulus, MD ^1^, Sahib S Khalsa, MD, PhD ^1,2^

^1^Laureate Institute for Brain Research, 6655 South Yale Ave, Tulsa, OK 74136

^2^Oxley College of Health Sciences, University of Tulsa, 800 S Tucker Dr, Tulsa, OK 74104

^3^Department of Psychology, University of Tulsa, 800 S Tucker Dr, Tulsa, OK 74104

^4^Stephenson School of Biomedical Engineering, University of Oklahoma, 202 West Boyd St. Norman, OK 73019.

# Supplementary Methods

## Participants

23 healthy individuals (22 right handed, 11 female; mean age: 26±6 years, body mass index: 25.9±4.3) participated in this study. These participants did not have any lifetime history of neurological, psychiatric, diabetic, cardiovascular or respiratory disorders, all were unmedicated, and all demonstrated normal vital signs, physical exams, and 12-lead electrocardiograms. No subjects were excluded due to adverse events. The study was approved by the Western Institutional Review Board and conducted at the Laureate Institute for Brain Research. All participants provided written informed consent and received compensation for their participation.

## Experimental Protocol

In order to parametrically modulate cardiorespiratory sensations and quantitatively measure the brain’s hemodynamic response to these changes, participants received intravenous bolus infusions of isoproterenol hydrochloride (1 or 2 micrograms (mcg) per dose; Valeant Pharmaceuticals, Laval, Quebec, Canada) during an ASL imaging session. Bolus infusions of normal saline were used as a control condition. Each dose (1mcg, 2mcg and saline) was repeated twice, resulting in a total of six infusion scans. Infusions were administered in a double-blinded manner at 60 seconds into each scan, and infusion order was randomized across subjects. Throughout each infusion scan (240 seconds/scan), participants continuously rated their experience of cardiorespiratory sensation intensity by rotating a MRI-compatible dial (Current Designs Inc., Philadelphia, PA) clockwise or counter-clock wise, with their dominant hand, when the sensations increased or decreased. They were instructed to keep their eyes open during the scans, and their dial ratings were displayed in real time on a computer screen in front of them (Supplementary Fig. S1). Concurrent with ASL recording, cardiac and respiratory waveforms were acquired at 40 Hz using a MRI-scanner equipped pulse oximeter and a respiratory transducer belt, and two MRI-compatible ECG leads (lead I and II configuration) were attached for continuous cardiac rhythm safety monitoring (GE Healthcare, Waukesha, WI, United States). Infusions were delivered by a nurse seated inside of the scanner room, with continuous visual access to all monitored vital signs. Immediately after each infusion scan, subjects were asked to verbally rate the intensity of experienced heartbeat and breathing sensations (0 = “none or normal” and 10 = “most ever”). To index affective state participants also reported the intensity of experienced positive emotion (“happy, excited, or euphoric”) and negative emotion (“anxious, tense or nervous”). Detailed instructions are as follows.

### Pre-Scan Training

A pre-scan training session was conducted with each participant approximately one hour before starting the fMRI scan. During this session participants were told they would be receiving both isoproterenol and saline infusions, at some point during the scan. They were informed what the isoproterenol sensations might feel like (e.g. “you may notice an increase in your heartbeat sensations, and/or may notice increase in your breathing sensations”). Because isoproterenol induces changes in both cardiac and respiratory signals participants were not given any instructions to focus preferentially on their cardiac or respiratory sensations, and each time the drug effects were described the experimenter made sure to describe potential cardiac and respiratory effects equivalently. During this discussion participants were encouraged to provide qualitative examples from their own life experience of analogous sensations. If they were unable to provide any they were given examples of high arousal situations such as physical exercise, riding a roller coaster, or presenting a speech at work, asked to imagine sensations occurring during them, and asked to verify whether they had ever experienced such sensations. All participants endorsed having experienced such sensations before in their lives.

Participants were told that during each infusion should continuously rate their overall experience of the intensity of cardiorespiratory sensations by rotating a dial clockwise or counterclockwise with their dominant hand when the sensations increased or decreased. The term ‘intensity of cardiorespiratory sensations’ was used with participants in relation to the dial ratings and explained as how noticeable they found those sensations to be overall, in each moment of rating. They were told to use the following anchor for their dial ratings: 0 = “none or normal” and 10 = “most ever” (similar to (Cameron and Minoshima, 2002; Khalsa *et al*, 2015; Khalsa *et al*, 2016; Khalsa *et al*, 2009a; Khalsa *et al*, 2009b)). Immediately after the infusion ended they were told they would be asked to individually rate the intensity of experienced heartbeat and breathing sensations using the same scale. They were told to also rate the intensity of experienced negative valence symptoms (i.e., “how anxious, tense, or nervous”) and positive valence symptoms (i.e., “how happy, excited, or euphoric”) using the same scale. We included several variants in terminology to account for possible individual variance in valence labeling, and included positive and negative valence items to avoid implicitly priming participants to one variant over another (as in (Khalsa *et al*, 2016)).

Participants were not informed when they would be receiving each agent (isoproterenol or saline), nor that there would be different doses (1 and 2 mcg). They were told only that they would verbally notified of the start of each infusion period (e.g., “infusion scan starting”) and that they would be asked to rate their experience after each infusion. During the training session, these verbal instructions were augmented by visual and written instructions presented on a computer screen (see ‘Participant instructions’ below). With the participant’s permission, we then administered a single saline infusion and a single isoproterenol infusion (1 mcg) while they were seated upright in an infusion chair, in counterbalanced fashion, and collected cardiorespiratory sensation ratings via dial and retrospective report. Immediately afterwards participants were informed that one of the infusions had contained the drug, but they were explicitly not given any feedback about which infusion contained the drug. We conducted these ‘practice infusions’ in order to 1) habituate participants to the infusion condition prior to scanner entry, and 2) to clarify any questions or confusion related to provision of cardiorespiratory sensation ratings prior to scanner entry. During the MRI scanning session, after each infusion scan verbal rating instructions were supplemented by visual and written instructions presented on a computer screen (see ‘Participant Instructions’ below).

### Participant Instructions

#### Rest Scan Instructions

- During the rest scan a small cross will appear in the center of the screen

+

- Keep your eyes open and focus on the cross
- Try to clear your mind and not think of anything in particular
- Do not fall asleep

#### Infusion Scan Instructions

- During each infusion scan you will receive an infusion of either isoproterenol or saline, shortly after the scan begins
- Before the infusion begins a message to “get ready” will appear
- Right before the infusion starts a countdown will begin, starting from 5

+

5

- The infusion will start when you see the words “infusion starting” appear

+

Infusion starting

#### Dial Rating Instructions

Throughout the scan you will use a dial to rate the overall intensity of your heartbeat and breathing sensations, on a scale from 0 (“none or normal”) to 10 (“most ever”)

- Only turn the dial above 0 if you feel an *increase* in your heartbeat and breathing sensations due to the infusion
- If you do not feel an increase in these sensations, please keep the dial at or below 0


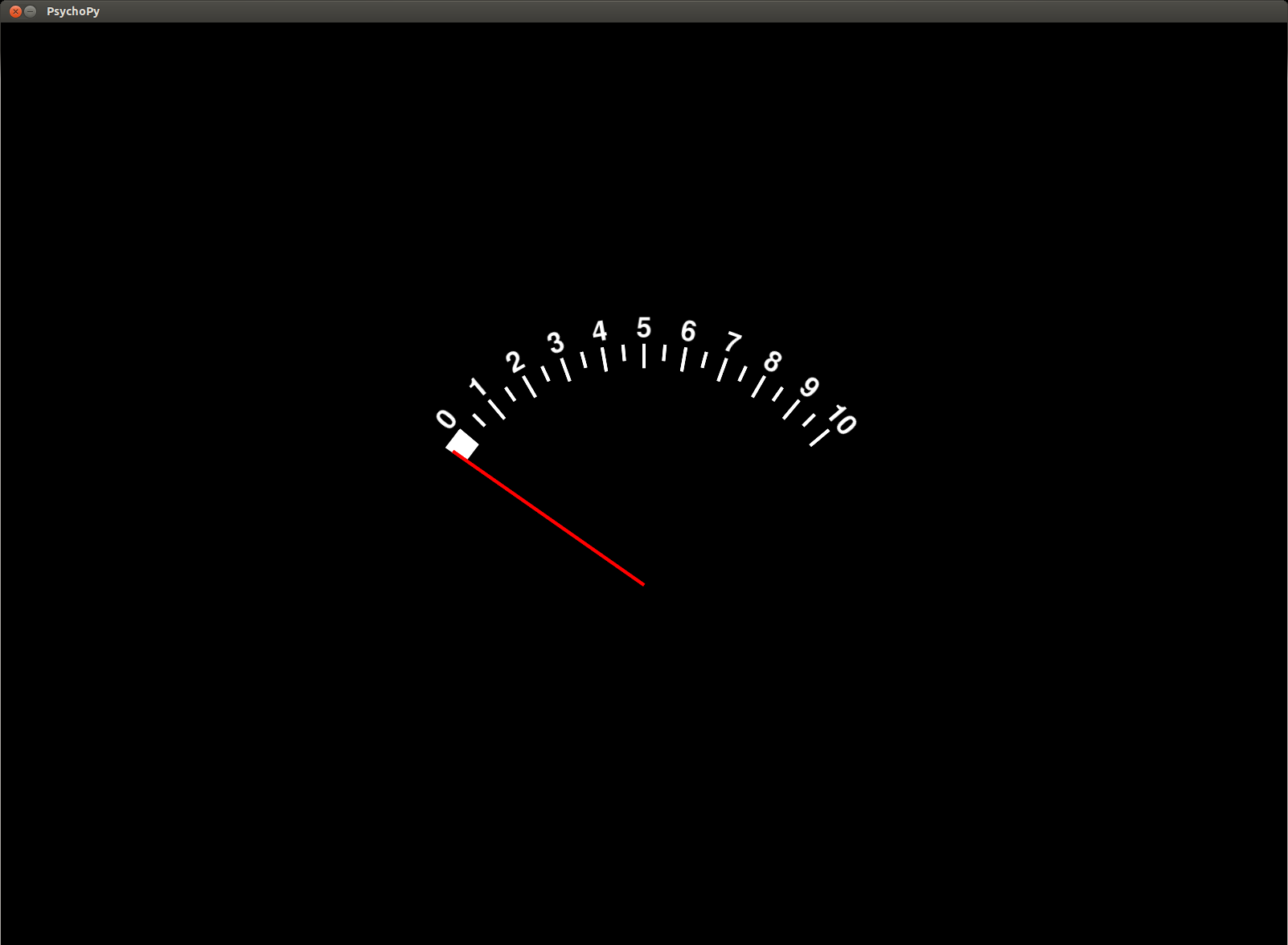

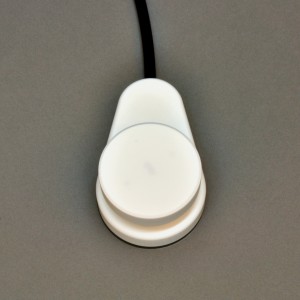


Retrospective Rating Instructions

After each scan we will ask you to make several ratings

- For ratings like these please give your response verbally

## MRI Data Acquisition

Experiments were performed on a 3 Tesla General Electric (GE) MR750 MRI scanner (GE Healthcare, Waukesha, WI, United States) with an 8-channel receive-only head coil. Head movement was minimized by securing the head with soft foam padding, and by lightly affixing a piece of soft tape to the participant’s forehead. A T1-weighted 3D MPRAGE sequence with FOV/slice thickness: 240/0.9 mm, *TR*/*TE*: 5.0/2.012 ms, *TD*/*TI*: 1400/725 ms, flip angle: 8°, 176 axial slices, SENSE *R*: 2, acquisition matrix: 256×256, sampling bandwidth: 31.2 kHz, scan time: 6 min and 13 s, was used for structural imaging. Functional images of 24 axial slices (recorded interleaved from bottom to top) were acquired using a pseudo-continuous ASL (pCASL) sequence and the following parameters: FOV/slice thickness: 220/5mm, matrix size: 64×64, labeling duration/post labeling delay: 1.8/1.5s, TR/TE: 4000/13ms, and flip angle: 90°. Two brief 12-second runs were collected at the end of the session to record minimum contrast and ASL calibration images to be used in cerebral blood flow (CBF) quantification.

## Data Analysis

***Pre-processing***: For each run, all functional volumes were co-registered to the first control volume and then spatially smoothed using 3D Gaussian kernel with full width half maximum of 6 mm in AFNI (Cox, 1996). To allow the MR signals to reach steady-state, the first 4 volumes were discarded. Time locked fluctuations in the respiratory and cardiac frequencies and their first harmonics were removed using the RETROICOR procedure (Glover *et al*, 2000; Restom *et al*, 2006) applied separately on tag and control images using a custom code in MATLAB (MathWorks Inc, Natick, Massachusetts, United States). Perfusion images were then calculated using pair-wise subtraction between control and label images and CBF values were calibrated. For calibration, first, all images including perfusion images, minimum contrast and ASL calibration images were co-registered to the subject’s T1-weighted structural image and then were brain masked. Imaging sensitivity profiles were estimated from minimum contrast images and used to correct perfusion images. ASL calibration images were also corrected for sensitivity and then normalized to the standard Talairach atlas (TT_Daemon) to calculate the mean CSF signal within the ventricles to be used as a measure of the equilibrium magnetization of arterial blood. CBF in each voxel was then calculated using a general kinetic model for pCASL (Alsop et al, 2015; Buxton et al, 1998). CBF images were then spatially transformed to the Montreal Neurological Institute (MNI) 152 atlas space using an affine transformation in FSL (Jenkinson *et al*, 2002).

***Statistical analysis:*** Subject-level maps of the brain response to different stages of cardiorespiratory stimulation were generated separately for each isoproterenol dose by using a block averaging method. Based on the experimental design, and our previous observations of different stages of group averaged heart rate changes and subjective dial ratings during isoproterenol infusions (Hassanpour *et al*, 2016; Khalsa *et al*, 2015; Khalsa *et al*, 2016; Khalsa *et al*, 2009b), we defined three time course blocks/periods: Baseline (0−60 s), Peak (80−140 s), and Recovery (160−240 s). Contrast maps for the peak (and separately, recovery) versus baseline periods were generated by subtracting the average of all the volumes within baseline blocks from those within peak (and separately, recovery) blocks. Next, group-level statistical maps of brain activation were generated by averaging the contrast maps across subjects and estimating the variance using a random effects analysis. Statistical maps were thresholded at p<0.005 (uncorrected). A cluster size analysis based on random field theory was performed to determine the statistical significance of above threshold cluster level family-wise error at p<0.05 (corrected).

# Supplementary References

Alsop DC, Detre JA, Golay X, Gunther M, Hendrikse J, Hernandez-Garcia L*, et al* (2015). Recommended implementation of arterial spin-labeled perfusion MRI for clinical applications: A consensus of the ISMRM perfusion study group and the European consortium for ASL in dementia. *Magn Reson Med* **73**(1): 102-116.

Buxton RB, Frank LR, Wong EC, Siewert B, Warach S, Edelman RR (1998). A general kinetic model for quantitative perfusion imaging with arterial spin labeling. *Magn Reson Med* **40**(3): 383-396.

Cameron OG, Minoshima S (2002). Regional brain activation due to pharmacologically induced adrenergic interoceptive stimulation in humans. *Psychosom Med* **64**(6): 851-861.

Cox RW (1996). AFNI: software for analysis and visualization of functional magnetic resonance neuroimages. *Computers and biomedical research, an international journal* **29**(3): 162-173.

Glover GH, Li TQ, Ress D (2000). Image-based method for retrospective correction of physiological motion effects in fMRI: RETROICOR. *Magnetic resonance in medicine : official journal of the Society of Magnetic Resonance in Medicine / Society of Magnetic Resonance in Medicine* **44**(1): 162-167.

Hassanpour MS, Yan L, Wang DJ, Lapidus RC, Arevian AC, Simmons WK*, et al* (2016). How the heart speaks to the brain: neural activity during cardiorespiratory interoceptive stimulation. *Philos Trans R Soc Lond B Biol Sci* **371**(1708).

Jenkinson M, Bannister P, Brady M, Smith S (2002). Improved optimization for the robust and accurate linear registration and motion correction of brain images. *Neuroimage* **17**(2): 825-841.

Khalsa SS, Craske MG, Li W, Vangala S, Strober M, Feusner JD (2015). Altered interoceptive awareness in anorexia nervosa: Effects of meal anticipation, consumption and bodily arousal. *Int J Eat Disord* **48**(7): 889-897.

Khalsa SS, Feinstein JS, Li W, Feusner JD, Adolphs R, Hurlemann R (2016). Panic Anxiety in Humans with Bilateral Amygdala Lesions: Pharmacological Induction via Cardiorespiratory Interoceptive Pathways. *J Neurosci* **36**(12): 3559-3566.

Khalsa SS, Rudrauf D, Feinstein JS, Tranel D (2009a). The pathways of interoceptive awareness. *Nat Neurosci* **12**(12): 1494-1496.

Khalsa SS, Rudrauf D, Sandesara C, Olshansky B, Tranel D (2009b). Bolus isoproterenol infusions provide a reliable method for assessing interoceptive awareness. *Int J Psychophysiol* **72**(1): 34-45.

Restom K, Behzadi Y, Liu TT (2006). Physiological noise reduction for arterial spin labeling functional MRI. *Neuroimage* **31**(3): 1104-1115.
