# Supplementary material for: The Insular Cortex Dynamically Maps Changes in Cardiorespiratory Interoception
Source: Neuropsychopharmacology. 2017 Aug 9;43(2):426–34. doi: 10.1038/npp.2017.154 (PMC5729563; doi:10.1038/npp.2017.154)
Supplement: Supplementary Results [file npp2017154x5.docx]

# **Full length Supplementary Results**

# The Insular Cortex Dynamically Maps Changes in Cardiorespiratory Interoception

Mahlega S Hassanpour, PhD ^1^, W Kyle Simmons, PhD ^1,2^, Justin S Feinstein, PhD ^1,2^, Qingfei Luo, PhD ^1^, Rachel Lapidus, BA ^3^, Jerzy Bodurka, PhD ^1,4^, Martin P Paulus, MD ^1^, Sahib S Khalsa, MD, PhD ^1,2^

^1^Laureate Institute for Brain Research, 6655 South Yale Ave, Tulsa, OK 74136

^2^Oxley College of Health Sciences, University of Tulsa, 800 S Tucker Dr, Tulsa, OK 74104

^3^Department of Psychology, University of Tulsa, 800 S Tucker Dr, Tulsa, OK 74104

^4^Stephenson School of Biomedical Engineering, University of Oklahoma, 202 West Boyd St. Norman, OK 73019.

# Supplementary Results

The activation maps shown in Figure 3 were based on data that were corrected for physiological noise using the RETORICOR method, which removes signal fluctuations that are time locked (synchronized) to cardiac and respiratory cycles. However, transient elevations in cardiorespiratory function are also known to induce non-synchronized physiological noise in fMRI data (Birn *et al*, 2008). We have recently shown that these non-synchronized noise artifacts can be effectively removed from ASL signals via a linear deconvolution model constructed using temporal gradients of cardiac and respiratory signals (Hassanpour *et al*, 2017). We evaluated whether adding this non-synchronized noise reduction method would (a) enhance CBF maps of the brain’s response to cardiorespiratory stimulation by strengthening observed activations, or (b) eliminate CBF changes related to brain activity because of non-synchronized temporal correlation with cardiorespiratory stimulation. Our analysis revealed a substantial increase in the volume of activated insula subregions during the peak period and to a lesser extent during the recovery period (Supplementary Fig. 3 Supplementary Tables 3 and 4), strongly supporting the former possibility. This additional physiological noise correction revealed another cluster of activation during the peak period, bridging the left precentral and postcentral gyrus in a location consistent with the hand region contralateral to the dominant hand. With this method we also detected a new cluster of activation during the recovery period in the same left medial frontal gyrus regions that was activated during the peak period (Supplementary Fig. 3, Supplementary Table 3).

# Supplementary References

Birn RM, Smith MA, Jones TB, Bandettini PA (2008). The respiration response function: the temporal dynamics of fMRI signal fluctuations related to changes in respiration. *Neuroimage* **40**(2): 644-654.

Hassanpour MS, Luo Q, Simmons WK, Feinstein JS, Paulus M, Luh W-M*, et al* (2017). Cardiorespiratory noise correction improves the ASL signal. *International Society for Magnetic Resonance in Medicine* **ISSN# 1545-4428**(0679).
