# Supplementary material for: The Insular Cortex Dynamically Maps Changes in Cardiorespiratory Interoception
Source: Neuropsychopharmacology. 2017 Aug 9;43(2):426–34. doi: 10.1038/npp.2017.154 (PMC5729563; doi:10.1038/npp.2017.154)
Supplement: Supplementary Tables [file npp2017154x6.docx]

# Supplementary Materials- Tables

**Supplementary Table 1:** Brain regions where CBF responses increased during the peak and recovery periods relative to the baseline period. A cluster defining threshold at p<0.005 (z = 2.58) and a cluster size threshold at p < 0.05 (clusters >70 voxels (560 mm^3^)) is applied to statistical maps. Cluster volume and the maximum z-value within each cluster and its MNI coordinates are shown.

| Contrast | Location | Volume (mm^3^) | Maximum z-value | MNI coordinate | | |
| --- | --- | --- | --- | --- | --- | --- |
|  |  |  |  | x | y | z |
| Peak  vs.  Baseline | Right mid-insula | 816 | 3.51 | 42 | 8 | -2 |
|  | Left medial frontal gyrus | 944 | 3.45 | -2 | -14 | 52 |
| Recovery  vs.  Baseline | Left mid-insula | 2248 | 4.06 | -36 | 6 | -2 |
|  | Right anterior mid-insula | 2072 | 3.98 | 34 | 8 | -2 |
|  | Right posterior insula | 1240 | 4.20 | 36 | -18 | 2 |

**Supplementary Table 2:** Breakdown of the percentage overlap for each cluster with respect to all probabilistic cytoarchitectonic insula subregions thresholded at 25% probability, as per Fan et al (2016). Percent overlap in a given subregion is relative to the cluster size, not the subregion itself. Due to the probabilistic nature of the atlas, parts of a cluster can overlap with multiple subregions. Consequently, the total percentage overlap values for each cluster do not sum to 100%.

| **Insula subregions** | **Peak vs. Baseline** | **Recovery vs. Baseline** | | |
| --- | --- | --- | --- | --- |
|  | Right mid-insula  (%) | Right anterior mid-insula (%) | Right posterior insula  (%) | Left mid-insula (%) |
| **hypergranular** | 0 | 0 | 61.3 | 0 |
| **dorsal granular** | 0 | 1.9 | 12.9 | 3.9 |
| **dorsal dysgranular** | 68.6 | 35.5 | 0.6 | 20.6 |
| **ventral dysgranular and granular** | 0 | 2.3 | 24.5 | 0 |
| **dorsal agranular** | 47.1 | 40.2 | 0 | 2.8 |
| **ventral agranular** | 0 | 10.4 | 0 | 1.8 |

**Supplementary Table 3:** Brain regions where CBF responses increased during the peak and recovery periods, after removing non-synchronized physiological noise. A cluster defining threshold at p<0.005 (z_th=2.58) and a cluster size threshold at p<0.05 (clusters >70 voxels (560 mm^3^)) is applied to statistical maps. The cluster volume and the maximum z-value within each cluster and its MNI coordinates are given.

| Contrast | Location | Volume (mm^3^) | Maximum z-value | MNI Coordinates | | |
| --- | --- | --- | --- | --- | --- | --- |
|  |  |  |  | x | y | z |
| Peak  vs.  Baseline | Right insula | 1216 | 3.8 | 42 | 8 | -2 |
|  | Left medial frontal gyrus | 1880 | 3.9 | -2 | -14 | 52 |
|  | Left precentral gyrus | 568 | 3.6 | -40 | -22 | 56 |
| Recovery  vs.  Baseline | Left mid-insula | 2016 | 3.9 | -36 | 6 | -2 |
|  | Right anterior mid-insula | 1856 | 3.8 | 40 | 8 | -2 |
|  | Right posterior insula | 1088 | 3.9 | 38 | -20 | 0 |
|  | Left medial frontal gyrus | 696 | 3.4 | -4 | -10 | 52 |

**Supplementary Table 4:** Breakdown of the percentage overlap for each cluster, after removing non-synchronized physiological noise, with respect to all probabilistic cytoarchitectonic insula subregions thresholded at 25% probability, as per Fan et al (2016). Note that these results are based on activation clusters after removing additional non-synchronized physiological noise. Percentages of overlap in a given subregion are relative to the cluster size, not the subregion itself. Due to the probabilistic nature of the atlas, parts of a cluster can overlap with multiple subregions. Consequently, the total percentage overlap values for each cluster do not sum to 100%.

| **Insula subregions** | **Peak vs. Baseline** | **Recovery vs. Baseline** | | |
| --- | --- | --- | --- | --- |
|  | Right mid-insula  (%) | Right anterior mid-insula (%) | Right posterior insula  (%) | Left mid-insula (%) |
| **hypergranular** | 0 | 0 | 55.9 | 0 |
| **dorsal granular** | 1.3 | 1.7 | 16.2 | 2 |
| **dorsal dysgranular** | 61.8 | 36.6 | 2.2 | 18.7 |
| **ventral dysgranular and granular** | 0 | 0.9 | 20.6 | 0 |
| **dorsal agranular** | 43.4 | 40.5 | 0 | 2.8 |
| **ventral agranular** | 0 | 8.6 | 0 | 2 |
