# Supplementary material for: The Insular Cortex Dynamically Maps Changes in Cardiorespiratory Interoception
Source: Neuropsychopharmacology. 2017 Aug 9;43(2):426–34. doi: 10.1038/npp.2017.154 (PMC5729563; doi:10.1038/npp.2017.154)
Supplement: Supplementary Figure Captions [file npp2017154x7.docx]

# Supplementary Materials – Figure captions

**Supplementary Fig. S1:** (A) Experimental protocol for ASL neuroimaging of cardiorespiratory interoceptive sensation during adrenergic stimulation with isoproterenol. (B) MRI-compatible dial used by participants to rate the intensity of their heartbeat and breathing sensations. (C) Top: visual display during the experiment. The red needle moved in real time to reflect intensity ratings on a scale from 0 (“none”) to 10 (“most ever”). Bottom: experimental timeline. At 45 seconds a visual cue (“Get ready”) appeared on the screen, followed by a visual cue signifying infusion onset at 60 seconds (“Infusion starting”). (D) After each infusion scan retrospective rating questions appeared on the screen along with a Likert scale.

**Supplementary Fig. S2:** Physiological changes induced by different levels of isoproterenol with respect to dial ratings (subjective experience of these changes). (A) Group averages of continuous heart rate (HR) (red curve) and cardiorespiratory intensity ratings (blue curve) during saline, 1 mcg, and 2 mcg isoproterenol infusions. A significant positive correlation between the peak heart rate increase and maximum dial rating was observed during isoproterenol infusion at 1 mcg (p=0.01) and 2 mcg (p=0.03) but not saline infusion (p=0.50) (B) Group averages of continuous respiratory rate (RR) (red curve) and cardiorespiratory intensity ratings (blue curve) during saline, 1 mcg, and 2 mcg isoproterenol infusions. Correlation between the maximum dial rating and RR increase during the peak period was not significant at 1 mcg (p=0.14) and 2 mcg (p=0.96) isoproterenol infusions but was significantly negative during saline infusion (p=0.02). (C) Group averages of continuous respiratory volume variability (RVV) (red curve), a derivative of respiratory signal, and cardiorespiratory intensity ratings (blue curve) during saline, 1 mcg, and 2 mcg isoproterenol infusions. Correlation between the RVV increase and maximum dial rating during the peak period was not significant at 1 mcg (p=0.12), 2 mcg (p=0.14) isoproterenol infusions or saline infusion (p=0.24).

**Supplementary Fig. S3:** Cortical activity during different stages of cardiorespiratory interoceptive processing following 2 mcg isoproterenol infusion before and after the additional removal of non-synchronized physiological noise. Clusters that appear after non-synchronized noise removal are circled.
